# Emergence of coronavirus disease 2019 (COVID-19) in Austria

**DOI:** 10.1007/s00508-020-01723-9

**Published:** 2020-08-20

**Authors:** Peter Kreidl, Daniela Schmid, Sabine Maritschnik, Lukas Richter, Wegene Borena, Jakob-Wendelin Genger, Alexandra Popa, Thomas Penz, Christoph Bock, Andreas Bergthaler, Franz Allerberger

**Affiliations:** 1 Österreichische Agentur für Gesundheit und Ernährungssicherheit (AGES), Spargelfeldstr. 191, 1220 Vienna, Austria; 2 Institut für Hygiene und Medizinische Mikrobiologie, Schöpfstr. 41, 6020 Innsbruck, Austria; 3 Institut für Virologie, Schöpfstr. 41, 6020 Innsbruck, Austria; 4 CeMM Research Center for Molecular Medicine of the Austrian Academy of Sciences, Lazarettgasse 14, 1090 Vienna, Austria

**Keywords:** Day of onset, Day of diagnosis, SARS-CoV‑2, Ischgl, Primary case

## Abstract

This is a report on the first identified cases of coronavirus disease 2019 (COVID-19) in Austria. The first documented case was a person who stayed in Kühtai, Tyrol, from 24 to 26 January 2020, and had been infected by a Chinese instructor in Starnberg (Germany) between 20 and 22 January. This counts as a German case since her diagnosis was eventually made in Munich (Germany) on 28 January. On 25 February, two cases imported from Italy were diagnosed in Innsbruck but again no secondary cases were identified in Austria. The first three infections of Austrian inhabitants were detected on 27 February in Vienna. The two resulting clusters finally included 6 (source of initial infection unknown) and 61 cases. Most likely, Italy was the source of the latter cluster. On 12 March the first fatal case of COVID-19 in Austria was reported, a 69-year-old Viennese who died in a Vienna hospital after returning from a cruise ship tour in Italy. On 6 March three autochthonously acquired cases were reported in the Tyrol, all related to the ski resort Ischgl. Of the first 14 Islandic COVID-19 cases infected in Ischgl, 11 had already returned to Iceland on 29 February. We consider that the incriminated barkeeper, who tested PCR positive on 7 March, was neither the primary case nor a superspreader. In our opinion, undetected transmission of SARS-CoV‑2 had been ongoing in Ischgl prior to the first laboratory confirmed cases. Our data also underline that the introduction of SARS-CoV‑2 into Austria was not one single event.

## Introduction

In December 2019, the severe acute respiratory syndrome coronavirus 2 (SARS-CoV-2) disease broke out in Wuhan, China [1]. Coronavirus disease 2019 (COVID-19), as it is named now, rapidly spread to other countries [2]. Cases in Europe initially were limited to small travel-associated clusters in Germany [3], France [4, 5] and the UK [6]. The outbreak was declared a public health emergency of international concern on 30 January 2020, and a pandemic on 11 March [7, 8]. In Austria, the emergence of COVID-19 is largely associated with the so-called Ischgl outbreak and often related as a superspreading event allegedly due to the barkeeper of an après ski bar [9]. This outbreak rapidly escalated by becoming a supranational epidemic. The primary aim of this study was to describe the first COVID-19 cases in Austria by time, place and personal characteristics, and to reconstruct their probable source of infection. A secondary aim was to illustrate the transmission dynamics of the first COVID-19 cases in Ischgl, a village with approximately 1600 inhabitants in the Paznaun valley in the Austrian state of Tyrol.

## Material and methods

The data presented include all initial patients in whom COVID-19 was documented by at least one nasal/pharyngeal swab specimen positive for SARS-CoV‑2 nucleic acid using a real-time reverse-transcriptase polymerase chain reaction (RT-PCR) assay in Austria. Data are according to the national registry epidemiological reporting system (Epidemiologisches Meldesystem, EMS) operated by the Austrian Agency for Health and Food Safety (AGES) on behalf of the Austrian Ministry of Health. Ethical approval was not necessary, as data collection and COVID surveillance are legal requirements. In Austria it became mandatory to report COVID-19 as of 27 January 2020 (regulation announced on 26 January 2020). For this report, patient data were de-identified. In Austria, the Department of Infection Epidemiology & Surveillance at AGES also has a legal mandate for contact tracing as part of the epidemiological investigation of the COVID-19 outbreak. Accumulations of cases within a certain time period in a certain region are called clusters. The aim of contact tracing is to rapidly identify potentially newly infected persons who may have come into contact with existing cases in order to reduce further onward transmission. By contact tracing, as of 29 May 2020, 5257 of the 17,034 patients who tested SARS-CoV‑2 positive, led to 355 epidemiologically defined clusters across the country. Contact tracing was performed according to technical guidance relating to this measure produced by the European Centre for Disease Prevention and Control (ECDC) [10].

## Results

### First patient

In Austria, the first COVID-19 patient was a 33-year-old female German citizen, who had stayed at the Alpine resort Kühtai (District of Imst) in Tyrol from 24 to 26 January 2020. She became ill on 24 January with acute respiratory symptoms (comorbidities none, initial symptoms very mild otitis, rhinitis and later symptoms hyposmia, hypogeusia) [11, 12]. For medical treatment, the febrile patient returned to Germany, where the COVID-19 diagnosis was confirmed by PCR on 28 January 2020. In accordance with ECDC surveillance criteria, the case was considered a German case. The diagnosis was confirmed microbiologically at the Institute for Microbiology of the Armed Forces (Institut für Mikrobiologie der Bundeswehr, InstMikroBioBw), Munich, Germany. This case #4 (numbering according to the date of positive PCR test results by the German public health authority) was one of four persons directly infected by a Chinese instructor who participated in several business meetings and workshops with different employees of an internationally connected automotive supplier in Starnberg, Bavaria, Germany; the index patient was symptomatic. Before the onset of symptoms, case #4 attended meetings with this Chinese index case at her company near Munich on 20–22 January. The business partner, a Shanghai resident, had arrived on 19 January 2020 from Shanghai (where she had probably been infected by her parents visiting from Wuhan) and flew back to China on 22 February 2020; she was tested positive for SARS-CoV-2 there on 26 January. Epidemiological investigations performed in Bavaria revealed case #4 as the source of one successive case in a coworker; no further cases occurred among the 23 contact persons staying with case #4 at the Alpine resort in Kühtai. Most likely China was the source country of this first COVID-19 patient’s infection in Austria. As of 25 May 2020, none of the 237 PCR-positive clinical specimens collected in Austria yielded the characteristic genomic pattern first described for the initial sample of case #4 [11, 12], disproving the hypothesis that the emergence of COVID-19 in Austria is linked to the so-called Munich cluster.

### First diagnosed cases

On 25 February, 2 COVID-19 cases (both 24 years old) tested PCR-positive for COVID-19 in Austria: both cases, an Italian couple working in Innsbruck, had returned from the Lombardy region (Italy) on 21 February. The female patient had onset of symptoms on 22 February and her partner on 24 February. Both were admitted to a hospital isolation ward on 24 February; microbiological diagnosis was performed at the Virology Institute of the Medical University of Innsbruck. The couple had had several contacts with sick people (relatives and friends) in their hometown over the entire week of their stay, they had also visited a big public event on 19 February. At the time they were tested for COVOD-19, no cases had been reported from their entire home region, although it then turned out to be one of the major hot spots in Italy. The female patient worked as a receptionist in an Innsbruck city hotel: none of the 59 contact persons (guests and staff) sampled during contact tracing was infected. On 20 February, the first autochthonous COVID-19 case in Italy was detected in a critically ill young man without known exposure to areas with viral circulation or known contact to a probable or confirmed COVID-19 case. All three previously diagnosed cases from Italy had a travel history to Wuhan [13]. As of 25 February, the World Health Organization (WHO) reported 229 confirmed cases in Italy. Italy was the likely source country of these first two SARS-CoV‑2 cases detected and confirmed in Austria.

### First cases diagnosed in Austrian residents

On 27 February, the first three Austrians with COVID-19 were diagnosed in Vienna. They belong to two epidemiologically unrelated clusters.

The so-called cluster Delta finally yielded six cases with unresolved source; there was neither an epidemiological link to other confirmed cases nor exposure to high-risk areas with ongoing person-to-person transmission detectable. Case #1 was sampled on 26 February, tested positive for SARS-CoV‑2 on 27 February at 09:20 am, and reported to the public health authorities at 09:33 am; microbiological diagnosis was performed at the Virology Institute of Vienna Medical University [14]. This first autochthonous SARS-CoV‑2 infection affected a 72-year-old lawyer operating an international law firm based in Vienna. He had been hospitalized on 17 February for flu-like illness and had developed pneumonia requiring ventilation treatment before being confirmed as a case of SARS-CoV‑2 infection. This case was detected during active case finding among approximately 1000 patients hospitalized in Viennese hospitals with severe acute respiratory tract infections, irrespective of any known exposure to a case or risk area of COVID-19. The active case finding strategy was initiated by the hospitals’ owner, the City of Vienna. As the criteria for testing at that time were limited to contact with a confirmed case or stay in a high-risk area, the patient had not initially been tested for SARS-CoV‑2. While none of 90 hospital staff members in contact with this case became infected, contact tracing among persons outside the hospital revealed 5 asymptomatic infections. The source of the infection in the index case remains unknown.

**Fig. 1:**
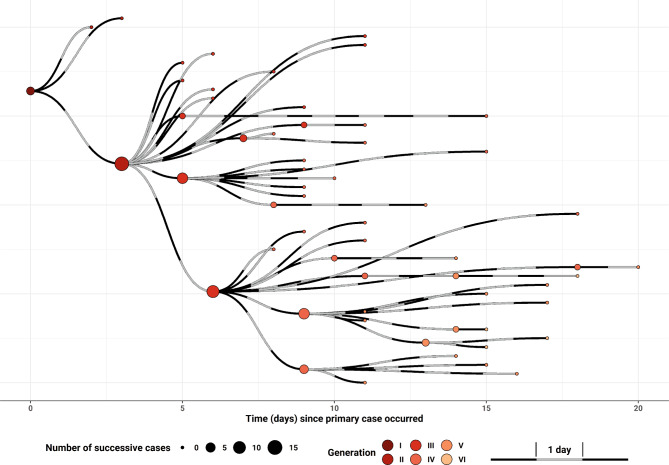
Transmission chain of COVID-19 satellite outbreak cluster A in the Austrian states Vienna and Lower Austria, February-March 2020 (created with R [http://www.R-project.org/], ggplot2 [https://ggplot2.tidyverse.org], ggraph [GraphPad Software, La Jolla California USA, www.graphpad].; data as of 16 April 2020)

The so-called cluster A included 61 cases (5 from Vienna, 54 from Lower Austria) with 6 generations of cases. Case #1, a 41-year-old male residing in Vienna, had an onset of mild respiratory symptoms on 23 February, the day he returned from a touristic visit to Milano (Italy). He was sampled on 26 February, tested SARS-CoV‑2 positive on 27 February at 8:42 am and was reported to the public health authorities at 14:37 the same day. His 46-year-old wife, case #2 was also sampled on 26 February, diagnosed on 27 February at 12:31 and reported at 14:29. Microbiological diagnosis was done at the Institute for Medical Microbiology Vienna of the Austrian Agency for Health and Food Safety (AGES). The patient reported onset of symptoms 3 days after her husband’s onset (26 February). The first two Austrian citizens with PCR confirmed COVID-19 infection were placed under quarantine in a Viennese hospital. All their known social contacts of the previous 4 days were tested. Their 15-year-old son, case #3, was sampled and PCR confirmed on 28 February; he had onset of symptoms the same day. In addition to these two family members, a female acquaintance (a self-employed fitness trainer who organizes indoor cycling classes), who had had dinner with the index case also turned out to be infected. Indoor cycling, often also called spinning, is a form of exercise with classes focusing on endurance, strength, intervals, high intensity (race days) and recovery, and involves using special stationary exercise bicycles in a classroom setting. The successive generations of this chain of transmission included a further 56 cases, 15 of them attendees of spinning classes (Fig. 1). In our opinion, any SARS-CoV‑2 infector conducting a spinning course can result in a superspreader event. Italy was the most likely source of this travel-associated SARS-CoV-2-cluster in Austria.

### First fatality in Austria

The first COVID-19 fatality in Austria occurred on 12 March 2020 in a hospital in Vienna: a 69-year-old Viennese citizen who was infected during a cruise ship tour in Italy (i.e. travel-associated case). The date of onset of his symptoms was 1 March; a nasopharyngeal swab was taken on 2 March. On 3 March, PCR diagnosis was confirmed at the Institute for Medical Microbiology Vienna of the Austrian Agency for Health and Food Safety (AGES).

### First cases related to Ischgl/Paznaun in Tyrol, Austria

**Fig. 2:**
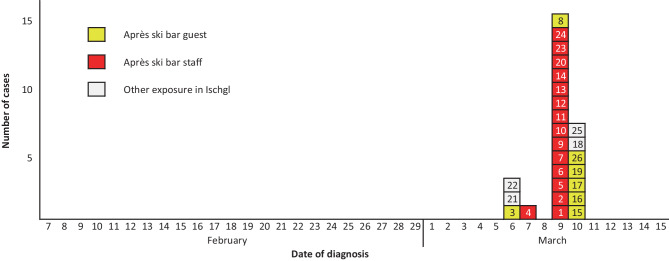
Cases related to Ischgl, reported until 10 March 2020, by day of laboratory diagnosis (*n* = 26)

The first locally acquired COVID-19 cases in Tyrol, all of them epidemiologically related to Ischgl (defined as residence or stay during a 14-day period to symptom onset), were PCR-confirmed on 6 March 2020, indicating ongoing local SARS-CoV‑2 transmission also outside eastern Austria. Using the date of laboratory diagnosis, these first three autochthonous cases were PCR confirmed in three Norwegian Erasmus students. They actively requested testing on 5 March after receiving a telephone call from a colleague who tested positive upon his return to Norway. As lschgl was not yet being considered a high-risk area, their skiing trip to Ischgl (28 February) remained initially unnoticed. On 8 March, the three students were contacted again by public health authorities and confirmed their stay at the ski-resort Ischgl. One of the three cases had visited the après ski bar X. All three were initially admitted to hospital quarantine and discharged to home isolation on 12 March. These Norwegian Erasmus students were previously travelling through Italy including stays in Lombardy. Not counting the two imported Italian cases from 25 February (City hotel in Innsbruck), the total number of confirmed cases increased from five cases on 6 March (including one case from another village, Pettneu outside Paznaun and one case in Kitzbühel imported from Italy, not shown in Fig. 1) to 25 cases on 9 March 2020 and 19 (76%) were epidemiologically connected to Ischgl. The aforementioned barkeeper was the first autochthonous case who visited a local physician, which explains why he was considered the index case by local public health authorities. His diagnosis was PCR-confirmed on 7 March at 19:13. Contact tracing and contact investigation, including extensive PCR-testing, was initiated on 8 March. On 9 March, 14 staff members and one guest of the après ski bar and on 10 March, 5 further après ski bar guests and 2 persons with exposure to Ischgl (but not to the bar) were identified as laboratory confirmed COVID-19 cases. These cases are presented in Fig. 2, which illustrates the cases by date of PCR diagnosis with their relevant features summarized in the Table 1. All cases were PCR confirmed at the Virology Institute of the Medical University of Innsbruck.

**Table 1:** Characteristics of COVID-19 cases related to Ischgl, as reported until 10 March 2020, by date of laboratory-diagnosis (*n* = 26)

ID	Sex	Age (years)	Country of origin	Date of onset of disease	Date of diagnosis	Exposure to après ski bar X	Home isolation or hospital admission	Intensive care treatment required	Place of residence when diagnosed	Comments
1	F	26	Austria	08.02.2020	09.03.2020	Staff	Isolation	–	Ischgl (Paznaun)	Waitress
2	M	50	Austria	27.02.2020	09.03.2020	Staff	Isolation	–	Ischgl (Paznaun)	Waiter
3	M	30	Norway	02.03.2020	06.03.2020	Guest	Hospitalized	–	Hall	Skiing in Ischgl (28 February)
4	M	35	Germany	02.03.2020	07.03.2020	Staff	Hospitalized	–	Ischgl (Paznaun)	Barkeeper
5	M	49	Austria	02.03.2020	09.03.2020	Staff	Isolation	–	Ischgl (Paznaun)	Disc jockey
6	M	25	Austria	04.03.2020	09.03.2020	Staff	Isolation	–	Ischgl (Paznaun)	Manager
7	F	25	Switzerland	05.03.2020	09.03.2020	Staff	Isolation	–	Ischgl (Paznaun)	Waitress
8	F	23	Switzerland	06.03.2020	09.03.2020	Guest	Isolation	–	Ischgl (Paznaun)	Close contact of case 7
9	F	24	Poland	06.03.2020	09.03.2020	Staff	Isolation	–	Ischgl (Paznaun)	–
10	M	43	Tunisia	07.03.2020	09.03.2020	Staff	Isolation	–	Ischgl (Paznaun)	–
11	M	23	Austria	07.03.2020	09.03.2020	Staff	Isolation	–	Ischgl (Paznaun)	–
12	F	30	Austria	07.03.2020	09.03.2020	Staff	Isolation	–	Ischgl (Paznaun)	–
13	M	52	Czech Republic	07.03.2020	09.03.2020	Staff	Isolation	–	Ischgl (Paznaun)	–
14	M	44	Slovakia	07.03.2020	09.03.2020	Staff	Isolation	–	Ischgl (Paznaun)	–
15	M	22	Sweden	07.03.2020	10.03.2020	Guest	Isolation	–	Ischgl (Paznaun)	–
16	M	21	Hungary	07.03.2020	10.03.2020	Guest	Isolation	–	Pfunds (Paznaun)	–
17	M	23	Austria	08.03.2020	10.03.2020	Guest	Isolation	–	Ischgl (Paznaun)	–
18	F	44	Austria	08.03.2020	10.03.2020	None	Hospitalized	Yes	See (Paznaun)	Local without Underlying disease
19	M	35	Austria	09.03.2020	10.03.2020	Guest	Isolation	–	Thaur	–
20	M	25	Austria	12.03.2020	09.03.2020	Staff	Isolation	–	Ischgl (Paznaun)	Manager and brother of case 6
21	F	25	Norway	–	06.03.2020	None	Hospitalized	–	Innsbruck	Skiing in Ischgl (28 February)
22	F	23	Norway	–	06.03.2020	None	Hospitalized	–	Innsbruck	Skiing in Ischgl (28 February)
23	F	40	Slovakia	–	09.03.2020	Staff	Isolation	–	Ischgl (Paznaun)	–
24	M	24	Germany	–	09.03.2020	Staff	Isolation	–	Ischgl (Paznaun)	–
25	F	67	Austria	–	10.03.2020	None	Hospitalized	Yes	Salzburg	Tourist with COPD
26	F	21	Czech Republic	–	10.03.2020	Guest	Isolation	–	Ischgl (Paznaun)	–

*F* female, *M* male, *COPD* chronic obstructive pulmonary disease

**Fig. 3:**
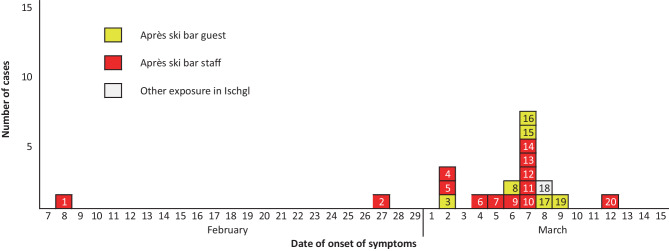
Cases related to Ischgl, reported until 10 March 2020, by day of symptom onset (*n* = 20)

In contact tracing, we usually rely on the date of onset of symptoms to identify infecting and infected persons in the chain of transmission (Fig. 3). A 26-year-old waitress (case #1) already showed signs and symptoms compatible with COVID-19 infection on 8 February. She suffered only mild symptoms, did not seek medical care and was working in the incriminated après ski bar in Ischgl. She was PCR-confirmed on 9 March, in the context of the active case finding among contact persons of the index case (barkeeper of bar X) mandated by the local public health authority. Several studies have demonstrated that SARS-CoV‑2 can be identified in throat and nasopharyngeal swabs even more than 60 days after onset of symptoms [15, 16] as might have been the case of the potential index case.

Case #2, a 50-year-old waiter from the same après ski bar, reported unspecific flu-like symptoms as of 27 February, which lasted for 10 days. Case #3 was a Norwegian Erasmus student who visited the après ski bar on 28 February and had onset of mild symptoms on 2 March. The same day, the barkeeper (case #4), and a disc jockey at the après ski bar (case #5) reported the onset of symptoms. Case #4 suffered from cough, fatigue and later developed fever and therefore consulted a physician; case #5 reported fatigue, muscle ache and low-grade fever. One staff member of the bar (case #22) reported onset of symptoms 4 days after the date of diagnosis (PCR diagnosed on 8 March and symptom onset on 12 March). For two further staff members (PCR diagnosed on 9 March), one guest (PCR diagnosed on 10 March) of the après ski bar and three persons with exposure to Ischgl other than the bar visit (all PCR diagnosed on 6 March) the exact date of symptom onset was not available. These cases are not included in Fig. 3, an outbreak curve based on date of onset of symptoms (case identification numbers as used in the Table 1).

Between 7 March and 17 March, a total of 145 COVID-19 cases were identified in and reported by the State of Tyrol, naming Ischgl as the place of residence (German *Wohnort, n* = 84) or current residence (German *Aufenthaltsort, n* = 61). Among those notified, two fatalities were registered (both native Tyroleans; 78-year-old male, 77-year-old female). With more than 22,000 beds for visitors and 1.4 million overnight stays during the winter season 2018/2019 Ischgl has the highest number of overnight stays per inhabitant in Tyrol [17]. More than 300,000 arrivals were reported during the winter season 2018/2019, with the average length of stay being 5 days [17].

On 6 March, three autochthonously acquired cases were reported in the Tyrol, all related to the ski resort Ischgl. We consider that the incriminated barkeeper, who tested PCR positive on 7 March, was neither the primary case nor a superspreader. In our opinion, undetected transmission of SARS-CoV‑2 had been ongoing in Ischgl for some time prior to the first laboratory confirmed cases.

On 3 March, an early warning and response system (EWRS) message from Iceland reported the first 3 COVID-19 cases with travel history to Austria. On 4 March, Iceland reported 8 cases probably exposed in Ischgl, and on 5 March the number of reported cases with travel history to Ischgl increased to 14. Of these 14 Islandic cases, 11 had already returned to Iceland on February 29: 1ne of them reported 26 February as the day of onset of symptoms, the others named days between 29 February and 3 March. Iceland reported that these COVID-19 cases were not travelling as one group, and the Islandic families had no known contacts among each other in Ischgl. Iceland declared Tyrol a high-risk area as of 29 February 2020 [18].

On 8 March, Denmark reported by EWRS 4 cases with exposure to Paznaun, the valley where Ischgl is located, and on 10 March 21 cases with exposure to the incriminated après ski bar. They had returned to Denmark the weekend of 7–8 March.

On 9 March, Norway sent an EWRS message, reporting 10 cases with confirmed, and 5 with likely exposure to Ischgl. On 8 February, Norway declared Tyrol as a place with ongoing transmission, and the Norwegian test criteria were revised to include exposure in Tyrol. From 8 March, there was a substantial increase in cases reported to have travelled to Austria, peaking with 176 new cases on 10 March. On 12 March, Norway reported 149 Norwegian COVID-19 cases with exposure to Ischgl.

Finland reported six cases with definitive exposure to Ischgl and included visits to Tyrol in the Finnish testing criteria on 9 March.

When the aforementioned barkeeper was confirmed positive on Saturday 7 March, the après ski bar was disinfected and newly staffed. The next day, Sunday 8 March, it reopened. Austrian health authorities finally closed all après ski bars in Ischgl by 10 March 2020, quarantining the village Ischgl and the whole valley of Paznaun on 13 March and banned all skiing in the Tyrol as of 14 March. Physical distancing orders came on 15 March when all 276 districts of Tyrol were locked down [19, 20].

Sequencing and uploading the genomic data of PCR-positive clinical specimens collected in Austria to the public database Global Initiative on Sharing All Influenza Data (GISAID) allowed comparison of the Austrian virus strains to all other publicly available SARS-CoV‑2 genomes [21]. The GISAID initiative involves public-private partnerships between the initiative’s administrative arm Freunde of GISAID e. V., a registered non-profit association, and governments of the Federal Republic of Germany, the official host of the GISAID platform, Singapore and the USA, with support from private and corporate philanthropic sources. The isolates of the Ischgl cluster matched the mutation profile of strains from cases in the French skiing resort of Contamines-Monjoue, in Haut-Savoie, where a British individual had travelled to France on 24 January, returning from a conference in Singapore (20–22 January), which was attended by 109 delegates, including one attendee from Wuhan (China) [22]; however, neither sequence information nor the results of our epidemiological investigation could identify a definite source of the SARS-CoV‑2 strain that led to the outbreak in Ischgl.

## Conclusion

Not since the emergence of the human immunodeficiency virus (HIV) in the 1980s has Austria been faced with an infectious diseases threat as serious as COVID-19. The daily life of every single person has been affected, although no complete lockdown was enacted in Austria. We are now advised to disinfect surfaces regularly, wash and dry hands, cough and sneeze into our elbows, not to touch our faces, to stay at home if we have a cold or flu-like symptoms and to call Healthline 1450. Ongoing contact tracing for all confirmed and probable new cases of COVID-19, with appropriate quarantine measures was put in place as well as testing of all suspected cases of COVID-19 for people meeting the currently valid definition criteria. Stringent self-isolation was implemented for those displaying relevant symptoms of COVID-19, those testing positive for SARS-CoV‑2, those who have a positive history of close contact with a confirmed case, including official quarantine/managed isolation for all those who have been abroad in the past 14 days. Quarantine facilities were implemented for those without sufficient capacity to effectively self-isolate. Robust border measures were put in place, which safeguard against the risk of COVID-19 being imported into Austria, with managed isolation or quarantine on arrival for 14 days before onward domestic travel. Due to these fundamental changes, it is not surprising that people ask for the patient zero, i.e. the person who introduced this new pathogen into the Austrian population; however, our data underline that the introduction of SARS-CoV‑2 into Austria was not one single event, which makes a patient zero unlikely.

In the case of Ischgl, the emergence of COVID-19 was assumed to be related to a superspreading event allegedly due to the barkeeper of an après ski bar [9]. Based on the facts presented in this article, we consider the barkeeper to be a scapegoat and not a superspreader; any overcrowded, noisy après ski bar, however, poses a perfect stage for superspreader events. In our opinion, silent transmission of SARS-CoV‑2 had been ongoing in Ischgl for some time prior to the first laboratory confirmed cases from clinical samples taken on 6 March. Crowding conditions in après ski bars harboring several infected staff members with mild symptoms during the influenza season probably led to an uncontrolled transmission. In cable cars, during queuing and on other premises where persons are in close contact to each other for longer periods as well as the weekly exchange of approximately 150,000 tourists in the Tyrol, usually on Saturdays, are also all likely to have contributed to the rapid and wide international spread. As of 12 May, Tyrol was the most affected province of Austria with 3518 confirmed cases and 107 fatalities. The district of Landeck, where Ischgl is located, reported the highest number of cases (*n* = 991, including active case finding within a PCR-based cross-sectional study) by district.

As a consequence of the nationwide spread of SARS-CoV‑2 in Austria, infection prevention and control measures have been implemented in many areas of public life to control local transmission. On 16 March the Austrian government announced a nationwide lockdown with restrictions on leaving home, which ended only on 1 May 2020; however, people not living in the same household still have to maintain a 1‑m distance from one another (on public transport this rule applies only where it is possible to have enough space). In certain enclosed spaces, for example in shops or while traveling on public transport, the mouth and nose still have to be covered. As of 11 May 2020, Austria has had 15,878 confirmed cases (179.2 per 100,000 population) with 622 fatalities (7 per 100,000 population). The estimated effective reproductive number of SARS-CoV‑2 spread in Austria has been continuously below one since 4 April. These numbers, in our opinion, suggest that Austria has coped fairly well with this pandemic; however, current recommendations, such as maintaining physical distancing of at least 1 m, wearing cloth face coverings if this is not feasible, washing hands often, covering coughs and sneezes, staying home when ill and frequently cleaning high-touch surfaces remain critical for reducing transmission. Molecular virus sequence analysis is expected to provide valuable additional evidence to complement the insights from contact tracing. A rigorous surveillance to identify, test and isolate all cases, and to trace and quarantine their contacts will be essential to ensure that localized outbreaks will not get out of control.
